# Post-Traumatic Stress Disorder and Related Factors in Parents of Children With Cancer in South-East of Iran

**DOI:** 10.5812/ircmj.2163

**Published:** 2012-12-06

**Authors:** Majid Naderi, Mahboubeh Firouzkoohi Moghadam, Mahdiyeh Hamzenejad, Abolfazl Emamdadi, Hossein Karami

**Affiliations:** 1 Children and Adolescent Hygiene Research Center (CAHRC) & Clinical Research Development Center (CRDC), Ali ebn-e Abitaleb (AS) Teaching Hospital, Zahedan University of Medical Sciences, Zahedan, IR Iran; 2 Bahran Psychiatry Teaching Hospital, Children and Adolescent Hygiene Research Center (CAHRC), Zahedan University of Medical Sciences, Zahedan, IR Iran; 3 Clinical Research Development Center (CRDC) & Medical Students' Research Committee (MSRC), Ali ebn-e Abitaleb (AS) Teaching Hospital, Zahedan University of Medical Sciences, Zahedan, IR Iran; 4 Thalassemia Research Center (TRC), Mazandaran University of Medical Sciences, Mazandaran, IR Iran

**Keywords:** Post-traumatic Stress Disorder, Parents, Malignant Neoplasm

## Abstract

**Background:**

Post-traumatic stress disorder (PTSD) comprises a collection of symptoms following exposure to injury-causing accidents of life. It is estimated that the prevalence of PTSD in children with malignancy and their parents is between 10-30% and even several years after treatment of malignancy this disorderremain in 20-20% of these patients.

**Objectives:**

This study investigated the prevalence of post-traumatic stress disorder in parents of children with cancer.

**Materials and Methods:**

In this analytic-descriptive study, 256parents of children with cancer (mean age: 30.06 ± 14.6 years-old) that their children treated in pediatric hematology ward of Ali ebn-e Abitaleb (AS) teaching hospital of Zahedan city (south east of Iran) at 2009-2010 were evaluated. The demographic data and symptoms of PTSD were collected by standard questionnaire (based on DSM-IV). After data analysis was performed using statistical software SPSS (version 18).

**Results:**

All parents who were studied had PTSD. The severity of PTSD in 111 of parents was mild, in 103 (40.2%) moderate and in 42 (16.4%) parents was severe. Furthermore, there were a significant correlation between the severity of PTSD with number of children, age of parents, gender, literacy, religion and economic state (P = 0.001).

**Conclusions:**

Our results showed that factors such as age, sex, number of children, educational state and religion of parents with economic state of the family can effect on the severity of PTSD. As for role of parents of children with chronic disease especially malignancy diseases on decline of psycho-social disorders with mental and physical supports of their children should be given the necessary recommendations and educations regarding PTSD.

## 1. Background 

Post-traumatic stress disorder (PTSD)comprises a collection of symptoms following exposure with injury-causing accidents of life; almost a person responses to this experience with fear and insolvency, continual depict that event in self-mind and also want to avoid the recall (1-3).The main clinical manifestations of PTSD are painful feeling of further incidence of undesirable experience, a pattern of emotional numbness and avoidance, and exceeding arousal feeling (3).Of course in DSM-IV-TR diagnostic criteria about PTSD had been listed that main symptoms of PTSD including of re-experiencing, avoidance and hyperarousal have lasted at least one month, and have impressed a serious effects on important areas of patient's life such as familial and social function (2, 3). Since child has been stood in a special position in the family, child care includes the entire members of a family, thereupon illness and emergency events related to the child can cause stress in the family, whether an acute/chronic or a mild/severe or a fatal disease (4).The lifetime prevalence of PTSD in the general population is about 8% and its incidence estimated 9-15%, that this prevalence is higher in women (10-12% *vs* 5-6% in males); this disorder may occurs at any age, but most common age of its onset due to modality of precipitator factors is in early adulthood (3). Malignant disease is one of the chronic diseases in children and most common death induced diseases especial in 10-12 years-old (1), and has been introduced as an important stressing factor in a family (2). Basically cancer in a child changes life status of his parents and necessitates difference encounters against it. Mothers implement major portion of cares and other services of her child that suffered from a malignant disease, thus they tolerate more stress and subject high psycho-social pressures versus their husbands (2). It is estimated that prevalence of PTSD in children with malignancy and their parents is between 10-30% and even several years after treatment of malignancy this disorder remain in 20-20% of these patients (5). during the treatment of a child cancer, their parents can be exposured with potential traumatic events such as diagnosis, training, observation of travail of their child, complaints of disease and its remedy, and the death of their child (6, 7). It is Based on the mentioned notes and limited studies in this setting special in south east of Iran (as for high prevalence of hematologic cancer in children).

## 2. Objectives

The aim of this study was to evaluate post-traumatic stress disorder (PTSD) and related factors in parents of children with cancer in south east of Iran at 2009-2010.

## 3. Materials and Methods

In this cross-sectional observational descriptive-analytical study, 256 parents (128 males and 128 females) of children with cancer (mean age: 30.06 ± 14.6 years-old) that their children were treated in pediatric hematology ward of Ali ebn-e Abitaleb (AS) teaching hospital of Zahedan city (south east of Iran) at August 2009- April 2010 were studied. The selection of samples was performed by simple random method and information was collected during 9 months. then the description of the necessary information regarding PTSD to interviews, early demographic information of studied persons including of: sex, age and literacy of parents, sex and age of their patient child, number of child, number of hospitalization of child, religion, family history of malignancy, family income was registered in the informational forms. Inclusion criteria for selection of parents were included:

1. At least 4 weeks had elapsed from diagnosis of malignant disease in their child.

2. In course of remedy patient child had owned no relapse period.

It should be mentioned that parents of the children with the following conditions had been excluded from our study while: death of child in duration of study, disinclination of parents for continuance of their child treatment. For survey of PTSD, was used the PTSD standard questionnaire according TO DSM-IV-TR that its validity had been confirmed by Mohammadi *et al* in Iran) was used (4). The questionnaire had 17 questions: 5 questions related to symptoms of re-experience (B), 7 questions related to symptoms of avoidance (C) and 5 questions relating to hyperarousal (D); that each studied parents for PTSD diagnosis should have at least one of symptoms of B and 3 of symptoms of C, with 2 of symptoms of D. For each answer of the questions, it was assigned (based on simple likert scoring method) a score from 0 to 3 (Non /Once a week or less, low or very low/2 to 4 times a week, occasionally or sometimes/more than 5 times per week and very much or almost always). For determining the severity of PTSD in the studied persons with scores more than 5 was used sum of the total scores of questions including: mild (5-13), moderate (14-20), severe (21-51) PTSD.Data analysis was performed by using statistical software SPSS (version 18) and Chi-square (X^2^) test was used to determine the correlation between quantitative variables (demographic data of studied persons in PTSD severity subgroups). Also Fisher Exact test was used as needed (for definition ofcorrelation between quantitative variables), by using a significant level of P < 0.05.

## 4. Results

In this study, of 256 parents of children with malignancy, 128 persons (50%) were male. The average age of studied parents was 30 ± 14.6 years-old (Age range: 29-75 years), the mean age in males was 32.6 ± 14.7 years-olds (Age range: 31-75 years); and among females was 27.2 ± 14.1 years-old (Age range: 29-70 years) that there was no correlation between genders (P = 0.056).It should be noted that 129 persons of studied parents (50.3%) had age lower 35 years, 111 persons (43.5%) aged 35-50 years and 16 persons (6.2%) had upper 50 years-old. Of 130 pediatric patients with malignancy, 87 patients (66.9%) were males and 43 patients (33.1%) female. In addition, 48 children (36.9%) lower 5 years, 47 children (36.1%) 5-10 years and 35 children (27%) had upper 10 years respectively. In studied patients children, 64 children (49.2%) leukemia, 28 children (21.6%) lymphoma, 38 children (29.2%) had solid tumor (Rhabdomyosarcoma, Wilms tumor and etc.). Of 64 children with leukemia, 34 children (53.1%) in the standard risk (SR), and 30 children (46.9%) were (46.9%) in the High risk (HR).Also, of 28 children with lymphoma, 8 children (28.6%) were in stage II of disease, 7 children (60.7%) in stage III and 3 children (10.7%) in stage IV. Among 38 children with solid tumor, 17 children (44.7%) were in stage II, 14 children (36.9%) in stage III and 3 children (10.7%) in stage IV disease. 85 parents (33.2%) were illiterate and in educational level, 75 persons (29.3%) were elementary school, 44 cases (17.2%) guide school, 49 cases (19.1%) high school/graduate and 7 persons (1.2%) were upper than graduate. Of 256 surveyed parents, 90 persons (35.1%) had 1-3 children, 84 persons (32.8%) 4-6 children and 90 persons (35.1%) had more than 6 children. The Number time of hospitalization of the patients children, in 125 parents (48.8%) was 2-5 times, 75 parents (29.3%) 6-10 times, 36 parents (14%) 11-15 times and 20 parents (7.9%) was Upper than 15 times. Only 21 studied persons (8.2%) had positive family history for malignancy. The levels of family income in 37.1% of studied persons were low income (lower 2,000,000 Iran Rials), and 46.4% moderate income (2,000,000-4,000,000 Iran Rials) and 16.5% had high income (upper 4,000,000 Iran Rials). All surveyed parents (100%) were Muslim, and for Islamic religion 109 persons (42.5%) Shiite and 147 persons (57.5%) were Sonnat. In our study, all assessed parents had some degree of PTSD, that the severity of PTSD in 111 cases (43.4%) was mild, 103 persons (40.2%) moderate and 42 persons (16.4%) severe. In 127 studied parents of patient child with leukemia, 56 persons (44.1%) had mild PTSD, 51 persons (40.2%) moderate PTSD and 20 persons (15.7%) severe PTSD. Also, in 56 studied parents of patient child with lymphoma, 24 persons (42.9%) had mild PTSD, 26 persons (46.4%) moderate PTSD and 6 persons (10.7%) severe PTSD. Of studied parents that had patient child with leukemia (73 persons), 31 persons (42.5%) had mild PTSD, 26 persons (35.6%) moderate PTSD and 16 persons (21.9%) severe PTSD (P = 0.486). in parents of children with stage II Solid Tumor/ Lymphoma, 25 persons (49%) had mild PTSD, 42 persons (43.2%) moderate PTSD and 4 persons (7.8%) severe PTSD; and among parents of children with stage III Solid Tumor/ Lymphoma, 24 persons (40%) had mild PTSD, 26 persons (43.3%) moderate PTSD and 10 persons (16.7%) severe PTSD. Also among parents of children with stage IV Solid Tumor/ Lymphoma, 7 persons (35%) had mild PTSD, 5 persons (25%) moderate PTSD and 8 persons (40%) severe PTSD. It Should be mentioned that among 65 studied parents of patient child with standard risk (SR) leukemia, 29 persons (44.6%) had mild PTSD, 28 persons (43.1%) moderate PTSD and 8 persons (12.3%) severe PTSD; while in 60 studied parents of patient child with high risk (HR) leukemia, 26 persons (43.3%) mild PTSD, 22 persons (36.7%) moderate PTSD and 12 persons (20%) had severe PTSD (P = 0.119). The distribution of PTSD severity in parents of child with malignancy based on the type and stage of malignancy in child has been shown, respectively in Table 1 and 2.

Furthermore, the distribution of PTSD severity in parents of child with malignancy based on the sex, age and literacy of parents, sex and age of child, number of child, number of hospitalization of child, Islamic religion, family history of malignancy and family income has been displayed in Table 3.

**Table 1:** Distribution of PTSD Severity in Parents of Child With Malignancy Based on Type of Malignancy in Child

Malignancy Type in Child	Mild, No. (%)	Moderate, No. (%)	Severe, No. (%)	Studied Persons, No.	*P* value
**Leukemia**	56(44.1)	51(40.2)	20(15.7)	127	0.486
**Lymphoma**	24(42.9)	26(46.4)	6(10.7)	56	
**Solid Tumor**	31(42.5)	26(35.6)	16(21.9)	73	

**Table 2:** Distribution of PTSD Severity in Parents of Child with Malignancy Based On Stage of Malignancy in Child

Malignancy Type in Child	Stage	Mild, No. (%)	Moderate, No. (%)	Severe, No. (%)	Studied Persons, No.	*P* value
**Solid Tumor and Lymphoma**	I	25(49)	42(43.2)	4(7.8)	51	0.119
	II	24(40)	26(43.3)	10(16.7)	60	
	IV	7(35)	5(25)	8(40)	20	
**Leukemia**	SR a	29(44.6)	28(43.1)	8(12.3)	65	
	HR a	26(43.3)	22(36.7)	12(20)	60	

^a^Abbreviations: HR, High Risk; SR, Standard Risk

**Table 3:** Distribution of PTSD Severity in Parents of Child with Malignancy Based on Demographic Futures

	Mild, No. (%)	Moderate, No. (%)	Severe, No. (%)	PTSD Severity
**Sex of Parents**				0.001
Male	79(62.7)	38(30.2)	9(7.1)	
Female	32(24.6)	65(50)	33(25.4)	
**Age of Parents, y**				0.001
Lower 35	45(34.9)	52(40.3)	32(24.8)	
35-50	55(49.5)	47(42.4)	9(8.1)	
Upper 50	11(68.7)	4(25)	1(6.3)	
**Literacy State of Parents**				0.001
Illiterate	56(65.9)	24(28.2)	5(5.9)	
Elementary School	32(44.4)	35(48.6)	8(7)	
Guide School	11(25)	23(51.3)	10 (22.7)	
High School/ Graduate	11(22.5)	20(40.8)	18(36.7)	
Upper Graduate	1(16.7)	1(16.7)	4(66.6)	
**Sex of Child**				0.486
Male	73(42.9)	70(41.2)	27(15.9)	
Female	38(44.2)	33(38.4)	15(17.4)	
**Age of Child, y**				0.053
Lower 5	39(41.5)	32(34)	23(24.5)	
5-10	39(40.6)	44(45.8)	13(13.6)	PTSD Severity
Upper 10	33(50)	27(40.9)	6(9.1)	
**Children, No.**				0.001
1-3	20(22.2)	39(43.3)	31(34.5)	
4-6	50(59.5)	27(32.2)	7(8.33)	
Upper 6	41(50)	37(45.1)	4(4.9)	
**Hospitalization of Children, No.**				0.543
2-5	48(38.4)	52(41.6)	25(20)	
6-10	35(46.7)	32(42.7)	8(10.6)	
11-15	17(47.2)	13(36.1)	6(16.7)	
Upper 15	11(55)	6(30)	3(15)	
**Islamic Religions**				0.001
Shiite	23(21.1)	51(46.8)	35(32.1)	
Sonnat	88(59.9)	52(35.4)	7(4.7)	
**Family History of Malignancy**				0.912
+	10(47.6)	8(38.1)	3(14.3)	
-	101(43)	95(40.4)	39(16.6)	
**Family Income**				0.001
Lower 2000000	52 (54.7)	35 (36.8)	8 (8.5)	
2000000-4000000	52 (43.7)	56 (47.1)	11 (9.3)	
Upper 4000000	7 (16.7)	12 (28.6)	23 (54.7)	

## 5. Discussion

PTSD prevalence have been reported 10-35% in previous studies (5, 8, 9). In our study, all surveyed parents (100%) had some degree of PTSD and the severity of PTSD in 111 cases (43.4%) was mild, 103 cases (40.2%) moderate and 42 cases (16.4%) severe. In a similar study that was performed in Philadelphia USA at 2004, PTSD had been diagnosed in 30% of mothers of children with malignancy and 13.7% of them had experience of treated PTSD, also in about 20% of studied families there was one parent with cured PTSD (10).In a study in 2005 at Children's Hospital of Philadelphia USA, were assessed 119 mothers and 52 fathers of 125 families that their children were treated for malignancy, 68% of mothers and 57% of fathers stood in the range of moderate to severe PTSD and in families that both parents were evaluated 38 persons (79.2%) were in the range of moderate to severe PTSD (6).A study in 2007 in USA showed that primarily in parents of children who had received active treatment compared with parents who had children with recurrent disease, fewer symptoms of PTSD were observed (11). In our study, only the parents of children who were receiving active treatment, were studied. In similar studies different risk factors for incidence and severity of PTSD symptoms have been reported (8, 9, 12). Based on the results of a study that was conducted in 2008 in Turkey, developing of PTSD is higher in female gender,and with following states including: better education levels, death one of dears, previous history of psychiatric disorders, a patient child with poor prognosis and use of radiotherapy (9). In 2006, Italy was performed a study on the mothers of children with malignancy that the results of this study showed that the age and gender of child, maternal education, parental occupation, socio-economic level have no effect on PTSD symptoms (8). According to the results of our study, there was no significant relationship between PTSD severity and type and stage of malignancy there was no significant relationship. But in a similar study in 2006 in Italy, the symptoms of PTSD in mothers of children with ALL were reported less than in parents of patients with AML (26.7% vs. 61.5%) and was observed a significant relationship between PTSD severity and type and stage of malignancy (8). The results of our study show whatever the age of parents be lower, the severity of PTSD is higher; and behalf severity of PTSD in mothers is more than fathers but there were no significant correlation between the severity of PTSD with age and gender of parents.In a study that accomplished in 2004, the severity of PTSD in young parents was more than elder parents (without significant correlation) (13); however in reliable references of psychiatry have implied elderly persons in exposure with stressors experience psychological pressures more than other persons, and prevalence of PTSD in females is further (3). The Mothers of children with chronic diseases especial malignancy have high sensitivity for affecting on psychiatric disorders and depression sensation, and always have delay in own goals and have more demand for appreciation and social supports (4). In similar studies on parents of children with malignancy in America (2005), Sudan and Turkey (2008), the severity of PTSD in women was reported more than men (6, 9, 14). Also in a study that was done in our center, the similar results were obtained in contrast with some studies, both parents of patient children had resembling levels of PTSD (10). In a similar study in Japan in 2006, PTSD was observed in 22.2% of fathers and 20.7% mothers (6). A similar study in Sweden in 2003 showed that the level of education had inversely relation with severity of PTSD, and PTSD with less intensity was observed in people with more education (2). But in our study was found that PTSD in parents with higher education levels is more severe than parents with less education. The study was conducted in Turkey in 2008 was reported the same results of our study (9); it can be concluded that parents with higher education levels due more awareness about their child's illness most obviously are affected by psychological stress. It should be mentioned that in our study was observed no significant relationship between income level and parental PTSD severity, but PTSD severity was greater in parents with higher income levels; Also in similar studies lower socio-economic level was reported as an effective factor on the severity of PTSD (5, 7) but some studies has been reported no association between this factor and PTSD (8).In our study all surveyed parents were Muslim, of course statistical analysis shows that PTSD in peoples with Shiite religion is more severe than peoples with Sonnat religion, that can be attributed this matter to more important of familial relationship in faith course of Shiite religion. The religion and faith factor has been assessed in limited studies; in a similar study in 2004 has pointed that religion can influence on severity of stress accidents (especially PTSD), so the subjective evaluation of life style and faith of patients are key factors for clinical assessment (5).In our study was observed that severity of PTSD in parents with less children was more than parents with more children, that can be attributed this matter to more interest and more wrong of parents with less number of children which is related to outcomes of disease and lose of own patient child. The effects of factors such as age and gender of the patient child, number of hospitalization of the patient children, having a positive family history for malignancy and number children of studied parents have not surveyed in other similar studies. The statistical data in our study were regarded that this demographic factors with severity of PTSD had no significant relationship.Accomplished Studies show that severe PTSD is related to risk factors of anxiety personality disorder, function of family, demographic conditions and different method treatment (7). Basically often PTSD that occurs with other psychological disorders is more severely and more likely to be chronic and may be difficult to treat. Access to social support may influence on onset, intensity and duration of PTSD. Patients that have good social relationship may not be substantially affected by PTSD or severe forms of this disease and also will be treating faster (3). Of course in our study, the other psychiatric disorders were not examined because of lack cooperation of studied persons. Our results showed that factors such as age, sex, number of children, educational state and religion of parents with economic state of family are affecting factors on the severity of PTSD.As for role of parents of children with chronic disease especially malignancy diseases on decline of psycho-social disorders with mental and physical supports of their children should be given the necessary recommendations and educations. Also, for more evaluation, more quantitative and comprehensive research has been suggested by evaluation of specific effective factors to psycho-social health of these patients.
